# The Effect of Game Importance on Concussion Incidence in the National Football League: An Observational Study

**DOI:** 10.7759/cureus.6252

**Published:** 2019-11-28

**Authors:** Theodore Hannah, Nickolas Dreher, Dhruv S Shankar, Adam Y Li, Jennifer Dai, Mark R Lovell, Tanvir F Choudhri

**Affiliations:** 1 Neurological Surgery, The Icahn School of Medicine at Mount Sinai, New York, USA; 2 Neurology, University of Pittsburgh Medical Center, Pittsburgh, USA

**Keywords:** football, concussion, national football league (nfl), head injuries, traumatic brain injury (tbi), sports medicine, american football

## Abstract

Introduction

Concussion incidence in the National Football League (NFL) has been shown to generally increase as the season progresses. Yet, there is evidence that suggests that the incidence stagnates or decreases in the final quarter of the season in comparison to the third quarter. This anomaly cannot be explained by any of the known modulators of concussion incidence. However, the fact that the teams start getting eliminated from playoff contention in the fourth quarter of the season may explain this pattern in concussion incidence. This study tests whether there is a difference in concussion incidence in games between teams who are still in the playoff hunt [in the hunt (IH) games] versus games where both teams have had their playoff fate already determined (non-IH games).

Methods

We obtained details of 166 documented concussions from weeks 13-16 of each of the four NFL seasons from 2012 to 2015 from Public Broadcasting Service's (PBS) Frontline Concussion Watch and matched them to the games in which they occurred. Each game was categorized based on the playoff status [clinched (CL), eliminated (EL), or IH] of the teams playing in the game. Concussion incidence of the game types was compared to each other using a one-way analysis of variance (ANOVA) test and student t-tests. Additionally, concussion incidences at six different player positions in important games were compared to the corresponding incidences in unimportant games. An ordinary least squares regression was used to examine the effects of game importance and plays per game on concussion incidence.

Results

Concussion incidence in important games (mean = 0.651 ±0.055) did not differ significantly (p: 0.890) from the incidence in unimportant games (mean = 0.623 ±0.143). Instead, plays per game was found to be the primary driver of concussion in the regression analysis (β = 0.01605; p: 0.025). At the position-specific level, running backs (RB) were the only position to demonstrate a significant increase in concussion incidence (p: 0.004) in important games (mean = 0.049 ±0.017) compared to unimportant games (mean = 0.00 ±0.00).

Conclusions

The results suggest that, in general, players are not more likely to suffer concussions in IH games than in non-IH games. However, RBs may have an increased risk of concussion in games with playoff implications than in games without.

## Introduction

Incidents of sports injuries have been shown to increase over the course of a game and over the course of a season [1-5]. This trend is often attributed to player fatigue. However, in the National Football League (NFL), there are previous reports of no change or a slight decrease in the incidence of some injuries, including concussions, from the third quarter (weeks 8-12) to the fourth quarter of the season (weeks 13-16) [1,6]. This stagnation of concussion incidence in the fourth quarter of the season is inconsistent with the cumulative fatigue hypothesis and cannot be explained by any of the other known modulators of NFL concussion incidence such as the strength of schedule, style of play, altitude, temperature, or dew point [7,8]. A novel modulator is necessary to explain this anomaly.

Studies have shown that a subject’s willingness to endure pain is modulated by the magnitude of the reward they expect to receive [9-13]. NFL players, having years of experience in making and receiving hits, are likely to be acutely aware as to which types of plays lead to which type of hits and which type of hits incur the most pain. Thus, players may modify their exposure to pain in accordance with their reward valuation of winning a game, and these adjustments in a player’s willingness to risk injury might modulate the incidence of concussion. 

The present study explores whether there is evidence to conclude that individual players increase their exposure to injury in games with playoff implications. Specifically, it tests the hypothesis that the concussion incidence is higher in games between teams who are still in the playoff hunt [in the hunt (IH) games] than in games where both teams have had their playoff fate already determined (non-IH games).

## Materials and methods

Data collection

The total number of wins accrued by each of the 32 NFL teams prior to weeks 13-16 of each of the 2012-2015 NFL seasons (four years) were collected from archived data from the NFL [14]. The number of wins of each team prior to each week’s games was used to sort the teams into three categories: IH, eliminated (EL), and clinched (CL). Teams were considered EL when they could no longer win enough games to be first in their division or catch the team in the second position in the wild-card race in their conference. Teams were considered to be CL if no other team in their division could match their wins or if they were in a top-two position in the wild-card race in their conference and the third-place team in the wildcard race could not match their wins. All other teams were assigned to the IH category.

Concussions occurring during the preseason or playoffs were excluded. Week 17 was also excluded because teams were not required to report concussions from that week if their team did not make the playoffs [15]. Analyses were restricted to weeks 13-16 after determining it was exceedingly rare for a team to be mathematically eliminated prior to week 13. Concussion data for weeks 13-16 of the 2012-2015 regular seasons were taken from Public Broadcasting Service's (PBS) Frontline Concussion Watch [16]. Frontline gives the following information about each concussion: player injured, player position, player team, number of games missed following the injury, and the week of the season the concussion was reported. All concussions were assumed to have occurred during games, although this is not always the case [2]. Each concussion (n = 166) was matched to the game (n = 256) with which it was associated.

Based on the classification of the teams playing (IH, EL, CL), each game was sorted into one of 6 categories: IH versus IH (IH v IH), IH versus CL (IH v CL), IH versus EL (IH v EL), CL versus CL (CL v CL), CL versus EL (CL v EL), and EL versus EL (EL v EL). The number of games in each category was as follows: IH v IH: 161, IH v CL: 17, IH v EL: 51, CL v CL: 4, CL v EL: 8, and EL v EL: 15. For analyses involving the fourth category, crucial (CR), teams that would have been sorted into the IH category were instead considered CR if they would be eliminated from playoff contention (as described above) should they not win that week’s game. This added four more game types: IH versus CR (IH v CR), CL versus CR (CL v CR), EL versus CR (EL v CR), and CR versus CR (CR v CR). And the number of games in each category was as follows: IH v CR:39, CL v CR: 4, EL v CR: 17, and CR v CR: 5. The adjusted number of games in each category was: IH v CR: 39, CL v CR: 4, EL v CR: 17, CR v CR: 5, IH v IH: 117, IH v CL: 13, IH v EL: 34, CL v CL: 4, CL v EL: 8, and EL v EL: 15.

For our position-specific analyses, concussion incidence at six positions was individually compared in IH games to non-IH games using the same protocol as described above. The positions analyzed were wide receivers (WR), running backs (RB), tight ends (TE), quarterbacks (QB), safeties (SA), and cornerbacks (CB). The corresponding number of concussions over 256 games was: 17 for WR, 13 for RB, 21 for TE, 5 for QB, 16 for SA, and 30 for CB.

Concussions per game for all analyses were calculated using the formula: number of concussions in relevant games/number of relevant games. Plays per game data were pulled from the sports data website TeamRankings (www.teamrankings.com) [17]. Plays per game were defined as the sum of total offensive plays for both teams reported by the TeamRankings website. Temperature and dew point data were taken from NFLWeather (www.nflweather.com) [18]. NFL stadium altitude data was obtained through ArcGIS NFL Stadium Elevation [19].

Data analysis

Data analysis was performed with Prism 8 (GraphPad, San Diego, CA). In accordance with statistical best practices to handle large differences in variance, all t-tests and one-way analyses of variance (ANOVA) were performed with Welch’s correction [20]. For all analyses, including correlations, t-tests, ANOVAs, and multilinear regressions, statistical significance was defined as α = 0.05. Mean values are presented with the standard error of the mean (SEM).

Regression analysis

A multivariate regression analysis to explain the number of concussions per games was performed using environmental variables known to affect concussions (altitude, temperature, and dew point), plays per game, and a dummy variable for importance of the game (IH dummy; 1 if at least one IH team is playing; 0 otherwise). The general form of the regression was: concussions = β0 + β1IH dummy + β2total plays + β3altitude + β4temperature + β5dew point.

## Results

**Figure 1:**
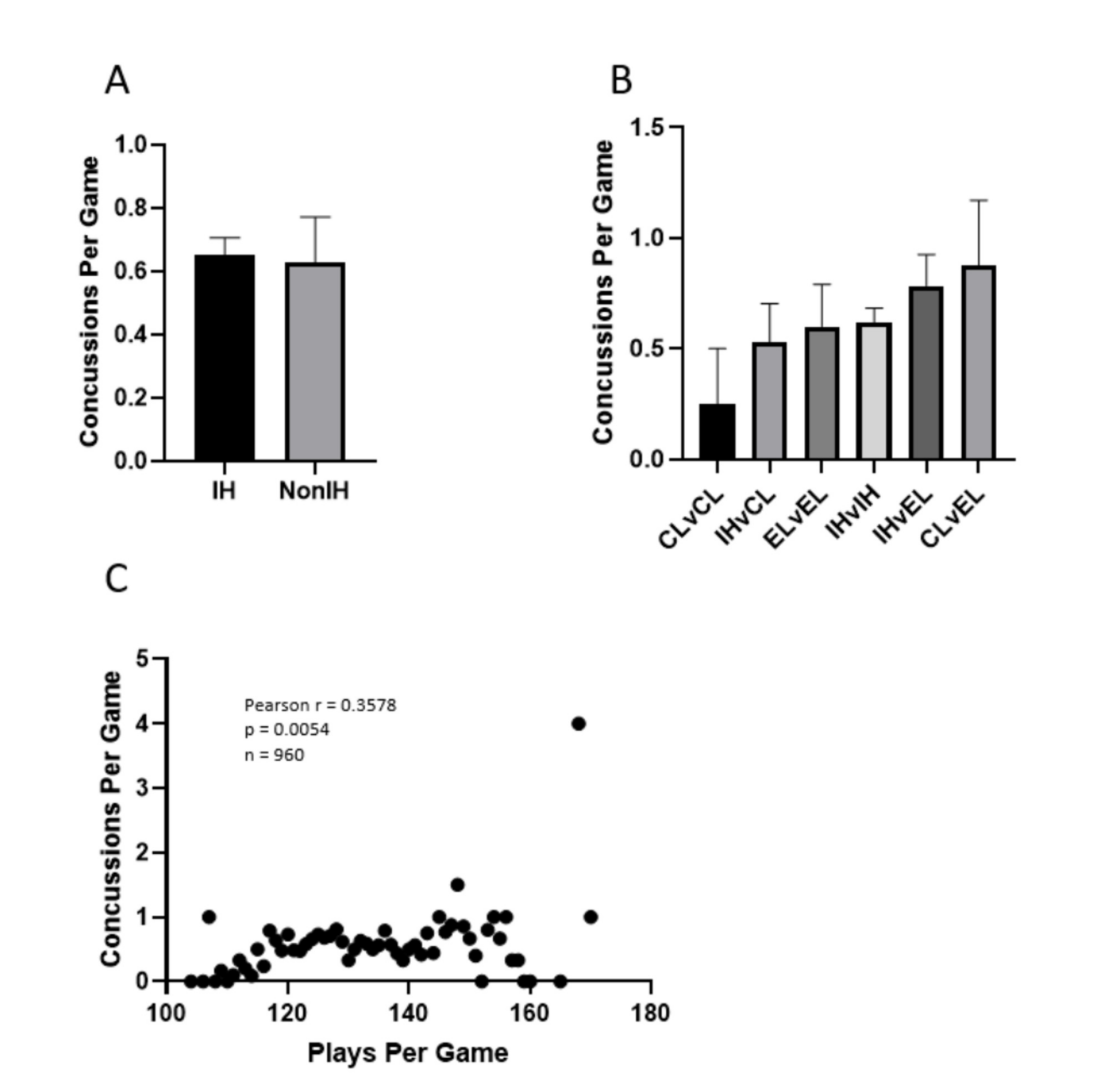
Effects of playoff implication on concussion incidence A) The number of concussions per game for games with at least one team in the playoff hunt (IH) versus games with no teams in the playoff hunt (non-IH). B) The number of concussions per game for each subdivided game type. C) The correlation between plays per game and mean concussions per game. All error bars represent SEM. No significant differences are present in either Figure 1A or Figure 1B CL: clinched; EL: eliminated; IH: in the hunt; v: versus; SEM: standard error of the mean

IH games (mean = 0.651 ±0.055) and non-IH games (mean = 0.623 ±0.143) were found to have similar concussion incidence (p: 0.890, Figure 1A). Concussion incidence was not significantly different between the six game types (IH v IH, IH v CL, IH v EL, CL v CL, CL v EL, and El v EL) [F(5,250): 0.693, p: 0.625], but there was a trend when teams in the same category play each other. Specifically, CL v CL games had the lowest concussion incidence (mean = 0.250 ±0.250) whereas EL v EL (mean = 0.600 ±0.1902) and IH v IH (mean = 0.6211 ±0.0623) games were both higher than CL v CL and similar in magnitude to each other (Figure 1B). Due to the non-significant results, the correlation between the number of plays per game and the concussion incidence was assessed. The correlation coefficient was positive and significant (r: 0.3578, p: 0.0054), but was moderate in magnitude (Figure 1C). We also found no significant differences in the average number of plays per game between the game types [F(5,250): 0.925, p: 0.466].

 

**Figure 2:**
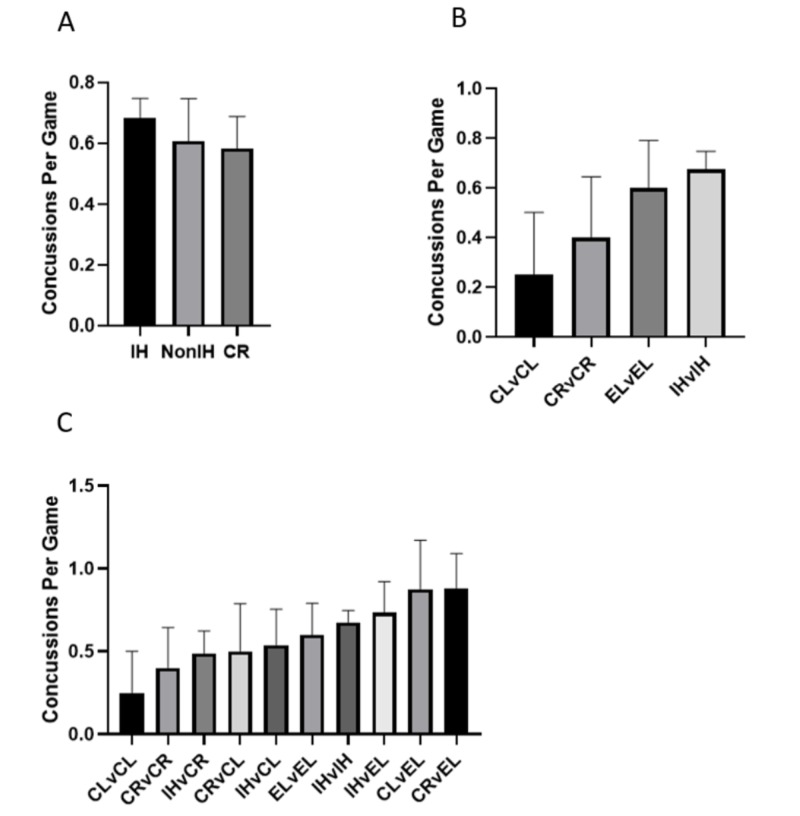
Effects of playoff implication on concussion incidence with separate elimination game category A) The number of concussions per game for games with at least one team in the playoff hunt (IH), no teams in the playoff hunt (non-IH), or at least one team facing elimination (CR).  B) The number of concussions per game for games in which teams of the same category play each other. C) The number of concussions for all subdivided game types. All error bars represent SEM. No significant differences are present in Figure 2 CL: clinched; EL: eliminated; IH: in the hunt; v: versus; SEM: standard error of the mean

To account for possible effects of a team being "pseudo-eliminated" (one loss away from playoff elimination), a new team category, CR, was created. However, even with this exclusion modification (Figure 2A), there was no statistical difference (p: 0.766) between games with at least one IH team (mean = 0.677 ±0.066) and games with only CL or EL teams (mean = 0.630 ±0.143). When these categories are broken down into specific game types (Figure 2C), no significant differences are found [F(9,246): 0.629, p: 0.772]. However, the trend in concussion incidence in games involving teams of the same category remained consistent (Figure 2B), with IH v IH games having the highest incidence (mean = 0.675 ±0.072), followed by EL v EL (mean = 0.600 ±0.190), CR v CR (mean = 0.400 ±0.245), and then CL v CL (mean = 0.250 ±0.250).

**Figure 3:**
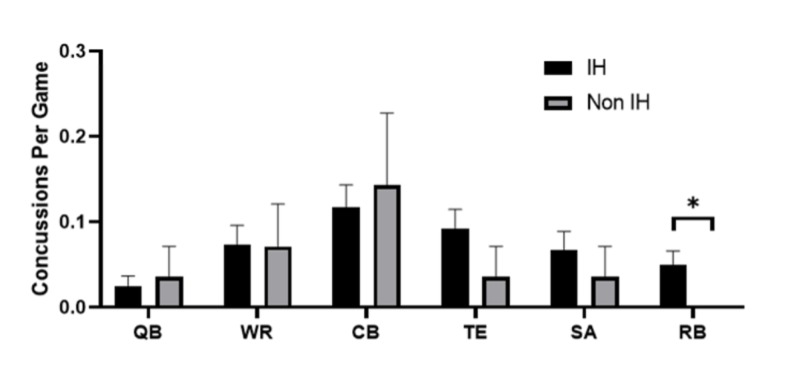
Position-specific effects of games with playoff implications The number of concussions per game across six different positions in games where at least one team is in the playoff hunt (IH) versus games with no teams in the hunt (non-IH). All error bars represent SEM QB: quarterback; WR: wide receiver; CB: cornerback; TE: tight end; SA: safety; RB: running back; SEM: standard error of the mean

Next, concussion incidence at a number of player positions during IH and non-IH games was examined. Figure 3B shows that RBs have significantly higher incidence of concussion (p: 0.004) in IH games (mean = 0.049 ±0.0170) compared to non-IH games (mean = 0.00 ±0.00) TEs appeared to follow this trend as well (IH mean = 0.092 ±0.0227, non-IH mean = 0.03571 ±0.0357), although the difference did not reach statistical significance (p: 0.189). WRs, QBs, SAs, and CBs all showed no difference in concussion incidence based on game importance. 

Finally, a regression analysis was performed using environmental modulators of concussion incidence, plays per game, and the importance of the game (Table 1). The results showed that the importance of the game does not significantly influence concussion incidence (p: 0.794), but plays per game do (p: 0.025), even when accounting for known environmental modulators. In this model, games with 10 more plays increased the number of concussions per game by 0.16.

**Table 1:** Regression analysis examining the relationship between concussion incidence and IH v non-IH games IH: in the hunt: games in which at least one team is in the playoff hunt; non-IH: games in which neither team is in the hunt; IH dummy: variable equal to 1 for IH games and equal to 0 for non-IH games; SE: standard error; ns: not significant

Explanatory variable	β	SE	t	P-value	Significance
Intercept	-1.406	0.9447	1.488	0.1384	ns
IH dummy	0.04426	0.1691	0.2617	0.7939	ns
Total plays	0.01605	0.007104	2.26	0.025	*
Altitude	-0.00003692	0.00006708	0.5504	0.5827	ns
Temperature	0.004438	0.006066	0.7317	0.4653	ns
Dew point	-0.005844	0.006084	0.9605	0.338	ns

## Discussion

To the best of our knowledge, this is the first study that has investigated the relationship between the importance of a game and concussion incidence. Surprisingly, the results suggest that, except for RBs, players are not more likely to suffer concussions in games with playoff implications than in games without. Instead, a major modulator of concussion risk is the number of plays per game [21].

Previous research has shown that TEs, RBs, CBs, and WRs have the highest rates of concussion, and passing positions overall have higher concussion incidence than running plays. This is attributed to the nature of collisions that occur in passing positions [1,22]. RBs did have higher concussion incidence in IH games, but contrary to expectations, the same effect was absent for TEs, CBs, and WRs. Due to the low sample size, further research will be necessary to validate and explain this phenomenon. However, one explanation is that there may be differences in fatigue and risk exposure inherent in various positions. For example, even though teams tend to throw the ball more often than they run it, an individual RB gets more touches per game than an individual receiver, increasing fatigue for the RB [7]. Furthermore, RBs get hit almost every time they touch the ball, increasing their exposure to concussion risk, whereas an individual WR gets fewer targets per game and gets hit on a lower percentage of those plays due to the prevailing rates of incomplete passes. 

The hypothesis for higher concussion incidence in IH games is predicated on the idea that players make riskier plays and/or increase the force of their tackles to increase their team’s chances of winning. The lack of significant differences both overall and at individual positions may be due to the naturally high-velocity impacts experienced at those positions accompanied by the highest concussion incidence. A previous study on concussion biomechanics demonstrated that a different concussion risk modulator, improved helmet manufacturing, can decrease concussion incidence for 16.6 mph and 20.8 mph impacts; however, for high-velocity impacts of 25.1 mph, the same improved helmet technology had zero effect on concussion incidence [23]. Since the player positions most vulnerable to concussion are also experiencing the highest velocity impacts, it may be more difficult for players to modulate their risk exposure than previously expected.

There were a few assumptions made in this study that, if untrue, may explain why the importance of the game does not appear to modulate concussion incidence. The first is that the effect of game importance on concussion incidence would be modulated solely through modifications that players make at the individual level. However, a previous study had concluded that a team’s style of play influenced concussion incidence [7]. Certain coaching styles may limit an individual player’s ability to modulate their exposure to concussion-inducing collisions. With a larger dataset, further stratification of games across the offensive and defensive styles of play of each team would likely be a valuable investigation. It was also assumed that the importance of the game to the team’s playoff hopes would be the single element of influence on how inclined a player would be to risk injury to improve their team’s chances of winning. However, players who are in the last year of their contract may be motivated to demonstrate their full potential irrespective of team standings. Moreover, when games become unimportant from a playoff standpoint, coaches may give second and third-string players opportunities to showcase their abilities to management to increase their stock for the following year. These players are much more likely to risk injury to prove their worth. Finally, all players were assumed to have an equal ability to adjust their games to reduce their risk of injury. It has been previously shown that players with lesser skills suffer concussions more frequently than more skilled players [24,25]. If players with lesser skills are less able to mitigate concussion risk but receive more playing time in non-IH games, this would dampen any effect of the skilled players avoiding concussions in non-IH games.

Limitations

In addition to the limitations inherent in these assumptions, this study is also limited by low sample size in many of the game-type categories of interest, leading to unequal variances between groups. A study that involves more seasons with reliable concussion data would improve the accuracy and power of these analyses. Additionally, without reliable data for week 17, the data set is restricted to less than 50% of all non-IH games played in a season. This introduces bias into the sample used since a large number of meaningful data points are missing. 

## Conclusions

The current study investigated the link between game importance and concussion incidence. Overall, concussion incidence does not appear to be affected by the playoff implications associated with each game. However, at the position-specific level, RBs are more likely to suffer a concussion in an IH game than in a non-IH one. This study also confirmed the number of plays per game influences concussion incidence. Future research should expand on this study with a more comprehensive dataset including concussions from week 17 and explore the interaction between plays per game and other known concussion modulators such as the style of play and strength of schedule.
